# Genome-wide identification of Hfq-regulated small RNAs in the fire blight pathogen *Erwinia amylovora* discovered small RNAs with virulence regulatory function

**DOI:** 10.1186/1471-2164-15-414

**Published:** 2014-05-31

**Authors:** Quan Zeng, George W Sundin

**Affiliations:** Department of Plant, Soil, and Microbial Sciences, Michigan State University, East Lansing, MI 48824 USA

**Keywords:** RNA-seq, sRNA, Type III secretion system, Biofilm, Amylovoran, Motility, ArcZ

## Abstract

**Background:**

*Erwinia amylovora* is a phytopathogenic bacterium and causal agent of fire blight disease in apples and pears. Although many virulence factors have been characterized, the coordination of expression of these virulence factors in *E. amylovora* is still not clear. Regulatory small RNAs (sRNAs) are important post-transcriptional regulatory components in bacteria. A large number of sRNAs require the RNA chaperone Hfq for both stability and functional activation. In *E. amylovora*, Hfq was identified as a major regulator of virulence and various virulence traits. However, information is still lacking about Hfq-dependent sRNAs on a genome scale, including the virulence regulatory functions of these sRNAs in *E. amylovora*.

**Results:**

Using both an RNA-seq analysis and a Rho-independent terminator search, 40 candidate Hfq-dependent sRNAs were identified in *E. amylovora*. The expression and sizes of 12 sRNAs and the sequence boundaries of seven sRNAs were confirmed by Northern blot and 5’ RACE assay respectively. Sequence conservation analysis identified sRNAs conserved only in the *Erwinia* genus as well as *E. amylovora* species-specific sRNAs. In addition, a dynamic re-patterning of expression of Hfq-dependent sRNAs was observed at 6 and 12 hours after induction in Hrp-inducing minimal medium. Furthermore, sRNAs that control virulence traits were characterized, among which ArcZ positively controls the type III secretion system (T3SS), amylovoran exopolysaccahride production, biofilm formation, and motility, and negatively modulates attachment while RmaA (Hrs6) and OmrAB both negatively regulate amylovoran production and positively regulate motility.

**Conclusions:**

This study has significantly enhanced our understanding of the Hfq-dependent sRNAs in *E. amylovora* at the genome level. The identification of multiple virulence-regulating sRNAs also suggests that post-transcriptional regulation by sRNAs may play a role in the deployment of virulence factors needed during varying stages of pathogenesis during host invasion by *E. amylovora*.

**Electronic supplementary material:**

The online version of this article (doi: 10.1186/1471-2164-15-414) contains supplementary material, which is available to authorized users.

## Background

*Erwinia amylovora* is a phytopathogenic enteric bacterium that causes fire blight, a devastating disease of rosaceous species such as apples and pears. Pathogen cells enter plants through the nectarthodes of flowers and other natural openings such as wounds, and are capable of rapid movement within plants and the establishment of systemic infections [1]. Many virulence determinants of *E. amylovora* have been characterized, including the Type III secretion system (T3SS), the exopolysaccharide (EPS) amylovoran, biofilm formation, and motility [2]. To successfully establish an infection, *E. amylovora* utilizes a complex regulatory network to sense the relevant environmental signals and coordinate the expression of early and late stage virulence factors involving two component signal transduction systems, bis-(3′-5′)-cyclic di-GMP (c-di-GMP), and quorum sensing [2–11].

One regulatory mechanism that has been utilized by many Gram negative bacteria to coordinate rapid responses to environmental signals is the use of regulatory small RNAs (sRNAs) [12–14]. sRNAs are a group of non-coding RNAs that are small in size, ranging from 50-350 nt, that target specific mRNA transcripts and control the translational efficiency and mRNA stability of the target mRNAs. Many sRNAs require the RNA chaperone Hfq for their stability and functional activation, and thus are referred to as the Hfq-dependent sRNAs [15]. Most of these Hfq-dependent sRNAs are encoded in the intergenic regions of bacterial genomes, with Rho-independent terminator sequences present at the 3’ end of the sRNAs. Once transcribed, the Hfq-dependent sRNAs are bound by Hfq, which stabilizes the sRNAs from degradation, and facilitates the interaction of the sRNAs to the target mRNAs [15].

Both computational and experimental approaches have been used in the identification of bacterial sRNAs. Computational predictions of bacterial sRNAs are generally based on the sequence conservation of sRNAs among closely-related species, the presence of orphan promoters and terminators in the intergenic regions of the chromosome, and conserved RNA secondary structure [16, 17]. Alternatively, many sRNAs have been identified by experimental methods, such as the shot-gun cloning method and tiling microarrays [18–20]. Recently, RNA-seq has revolutionized transcriptome analysis and led to the identification of numerous sRNAs in many bacterial species, including in a few plant pathogenic bacteria [21–23].

Bacterial sRNAs have been extensively studied in *Escherichia coli*, with more than 100 sRNAs identified and some of the functions of sRNAs characterized [24–26]. Although many sRNAs are conserved across bacterial species, many others are species specific and require experimental characterization in individual species. For example, among a total of 93 sRNAs identified in *Salmonella enterica*, only 45 of them were conserved in *E. coli*, and the other 48 are *Salmonella-*specific sRNAs [27]. Similarly, 32 sRNAs were identified in *Yersinia pestis*, among which only 14 sRNAs were also conserved in *E. coli*[28]. In *Pseudomonas aeruginosa*, 500 novel sRNAs were identified by RNA-seq, and nearly 90% of these novel sRNAs had no orthologs in other bacterial species [29].

Among all the sRNAs discovered in bacterial pathogens, many of them are involved in virulence regulation [30]. For example, in *Yesinia pseudotuberculosis*, mice infected with the deletion mutants of two sRNAs (Yrs35 and Yrs29) showed significantly increased survival rates and decreased weight loss in comparison to mice infected with the wild type *Y. pseudotuberculosis*[28]. In *Vibrio cholerae*, Qrr1-4, TarA, TarB, and VrrA control virulence by regulating cholera toxin (CT) biosynthesis and the toxin-co-regulated pilus (TCP), the two primary virulence factors that are responsible for colonization and subsequent disease development [31]. TarB and VrrA directly bind to the 5’ UTR and control the expression of the *tcp* genes [32] whereas Qrr1-4 indirectly regulate CT and TCP by controlling the quorum sensing regulators HapR, LuxO, and transcriptional activator AphA [33–35]. In *S. enterica* Typhimurium, four sRNAs contribute to bacterial pathogenesis [30], including MgrR which regulates *eptB*, the modulator of LPS modification [36], InvR that represses *ompD*, encoding the outer membrane protein synthesis protein [37], and SgrS that controls *ptsG* and *sopD*, two genes involving in sugar uptake and regulation of secreted virulence factor [38]. In the plant pathogen *Xanthomonas campestris* pv vesicatoria (Xcv), deletion of the sRNA sX12 resulted in reduced virulence in infected pepper plants [21]. In our recent work, we observed that in *E. amylovora*, the deletion of the sRNAs ArcZ (RyhA) and RprA significantly reduced disease symptoms in an immature pear infection model [39].

In addition to the sRNAs, the function of Hfq as a regulator of virulence traits has been demonstrated in many animal and plant pathogenic bacteria [40]. For example, in *S. typhimurium*, an *hfq* mutation resulted in highly attenuated invasion in epithelial cells and a significant reduction in growth in both epithelial cells and macrophages *in vitro*[41]. Multiple aspects of virulence were controlled by Hfq, including motility, outer membrane protein production, and intracellular growth. In *Agrobacterium tumefaciens*, the mutation of *hfq* led to reduced tumor formation on potato tubers, as well as delayed growth, altered morphology, and reduced motility. The uptake systems and metabolic versatility of *A. tumefaciens* were also tightly controlled by Hfq [42]. In our recent work, we identified Hfq as a regulator of virulence traits in *E. amylovora*, including the T3SS, amylovoran EPS production, biofilm formation, and motility [39]. These observations suggest that Hfq along with sRNAs regulated by Hfq has a critical function in virulence regulation in various bacterial pathogens including *E. amylovora.*

Although 10 sRNAs were predicted in *E. amylovora* by sequence conservation [39], the full repertoire of sRNAs of *E. amylovora* has not been experimentally characterized. We hypothesized that we could utilize RNA-seq and bioinformatic approaches to identify additional sRNAs in *E. amylovora*, and potentially identify novel sRNAs that regulate virulence in this plant pathogen. To acquire a better understanding of the Hfq-dependent sRNAs in *E. amylovora* and their roles in virulence manipulation, we conducted a genome-wide identification of Hfq-dependent sRNAs by combining Illumina deep sequencing, bioinformatics terminator prediction, and experimental validation by 5’ RACE and Northern blot. A total of 40 candidate Hfq-dependent sRNAs were identified in the genome of *E. amylovora*, many of which were novel sRNAs and *Erwinia-*specific sRNAs that were identified for the first time. In addition, four sRNAs, ArcZ, RmaA (Hrs6), OmrAB, and Hrs21, were identified as regulators of different virulence phenotypes during *E. amylovora* pathogenesis.

## Methods

### Bacterial strains, plasmids, and culture conditions

The bacterial strains, plasmids, and primers used in this study and their relevant characteristics are listed in Additional file 1: Table S1. All strains were stored at –80°C in 15% glycerol and cultured in Luria Bertani (LB) or Hrp-inducing minimal medium [43] at 28°C. When required, antibiotics were added to media at the following concentrations: gentamicin, 15 μg ml^-1^; chloramphenicol, 30 μg ml^-1^; kanamycin, 50 μg ml^-1^; and ampicillin, 100 μg ml^-1^.

### RNA isolation and RNA-seq

*E. amylovora* Ea1189 and Ea1189Δ*hfq* were cultured in LB broth for 14 hr. Cells were pelleted by centrifugation, washed with 0.5 X PBS, and resuspended in Hrp-inducing minimal medium with the optical density OD_600_ adjusted to 0.5. At 6 hr post-inoculation, RNA protect (QIAGEN; Valencia, CA) was added to the cell suspension to stabilize the cells. Total RNA was isolated using the miRNeasy Mini kit (QIAGEN), and DNA was removed by an on-column digestion of RNase-Free DNase (QIAGEN) during the RNA isolation. The quantity and quality of RNA was analyzed using a NanoDrop™ 1000 spectrophotometer (NanoDrop Technologies, Inc.; Wilmington, DE) and 2100 Bioanalyzer (Agilent Technologies; Santa Clara, CA). Total RNAs that passed quality control were used to generate sRNA libraries using the Illumina TruSeq small RNA sample preparation kit (Illumina; San Diego, CA). One microgram of total RNA from each sample was used for the library construction. Library molecules were amplified with 11 cycles of PCR. Fragments from 145-400 bp were isolated from the gel for further analysis. RNA-seq analysis was performed at the Michigan State University Research Technology Support Facility (MSU RTSF) using an Illumina HiSeq 2000 system.

### Reads mapping and identification of sRNAs in the *E. amylovora* genome

Reads that passed filtering were mapped to the *E. amylovora* ATCC 49964 genome [44] using TopHat (v2.0.4; http://ccb.jhu.edu/software/tophat/index.shtml). Filtered alignments of the *E. amylovora* genome were used as input to Cufflinks (v2.0.2; http://cufflinks.cbcb.umd.edu/manual.html) to identify intergenic regions for which the expression was significantly reduced in Ea1189Δ*hfq* (6 hr and 12 hr incubation) compared to Ea1189 (6 hr and 12 hr incubation), respectively. Statistically-significant differentially-expressed sequences from intergenic regions were obtained by applying a cutoff threshold of FDR ≤ 0.05 (5%) and an absolute log_2_ fold-change ≥ 0.6. The candidate transcript models were further manually inspected using the Artemis genome browser [45] to exclude regions whose transcription resulted from an extension from the adjacent ORFs. The remaining transcript models were annotated as the Hfq-dependent sRNAs. Per base depth was calculated by counting the number of reads overlapping each position of the *E. amylovora* genome, after excluding reads which overlapped known tRNA and rRNA loci. Counts represent the aggregate for mapped reads from all replicates of each condition. Depth for each condition was normalized per million reads aligned from each condition (excluding tRNA, rRNA overlapping reads). No duplicate removal was done for this study. The raw data and processed data were uploaded to the NCBI GEO database (http://www.ncbi.nlm.nih.gov/geo/) with the accession number GSE53763.

### Rho-independent terminators search

The intergenic regions of *E. amylovora* ATCC 49964 genome were analyzed by Python script (https://github.com/alejorojas2/Common_scripts/blob/master/Upstream_Ea.py), with the purpose of identifying sequences that meet the following criteria simultaneously: (1) containing at least 6 oligo-Us at the 3’ end; (2) containing at least 4 G + Cs in the last 6 nucleotides immediately upstream of the oligo-Us; (3) containing at least 50% G + Cs in the last 25 nucleotides immediately upstream of the oligo-Us. The sequences that met these criteria were further analyzed by CLC Main Workbench version 6.5 (CLC Bio, Denmark), for RNA secondary structure. Sequences that contain stem-loop structure with the free energy ∆G < 5.0 kcal mol-1 were further manually checked for the upstream transcriptional activity in Artemis genome browser. Sequences that showed upstream transcription were documented as potential Rho-independent terminators.

### Northern blot analysis and 5’ RACE assay

Northern blot of sRNAs was performed as previously described [46]. Briefly, 10 μg of total RNA was analyzed on a 6 M urea/6% polyacrylamide gel using a Northern Max kit (Life Technologies, Grand Island, NY) according to the manufacturer’s instructions. Hybridization probes were synthesized and 5'labeled by Life Technologies. Signals were developed using the Bright-Star BioDetect kit (Life Technologies). 16S rRNA was visualized under UV transilluminator (Syngene, Frederick, MD, U.S.A.) and used as an internal control for normalization of RNA. A biotin labeled Low Range ssRNA Marker (New England BioLabs) was used to determine the sizes of sRNAs.

5’ RACE assays were performed as previously described [39]. Briefly, 12 micrograms of total RNA from *E. amylovora* Ea1189 was treated with tobacco acid pyrophosphatase (Epicentre, Madison, WI) at 37°C for 0.5 h. 300 pmol of RNA oligonucleotide linker (GACGAGCACGAGGACACUGACAUGGAGGAGGGAGUAGAAA) was added to the treated RNA. RNA-linker mix was purified by phenol-chloroform-isoamyl alcohol (P-C-I) extraction and ethanol/sodium acetate precipitation method and was dissolved in 14 μl of RNase-free H_2_O. Purified RNA-linker mix was denatured at 90°C for 2 min and was ligated by T4 RNA ligase (New England BioLabs). Buffer and enzyme were removed by P-C-I extraction again and the ligated RNA-linker was dissolved in 10 μl of RNase-free H_2_O. cDNA was synthesized by SuperScript III reverse transcriptase (Invitrogen, Carlsbad, CA) using random hexamers following the instructions of the kit. The 5’ end of target sRNAs was amplified by PCR using the total cDNA as the template, RNA linker primer as the forward primer and primers specific for the target genes as reverse primers. The amplified PCR products were visualized on an agarose gel. Bands with the largest size whose intensity was enhanced in the tobacco acid pyrophosphatase treated samples compared to the non-treated samples were excised, purified and sequenced to determine the 5’ ends of the transcripts.

### Nucleotide conservation analysis of *E. amylovora* sRNAs

Sequences of candidate sRNAs obtained from the RNA-seq experiment were used in a Blast search against the genomes of 20 γ Proteobacteria in the ASAP database (https://asap.ahabs.wisc.edu/asap/logon.php). The nucleotide identity as well as the nucleotide length of the candidate sRNAs was obtained from the Blast search. The nucleotide conservation score was calculated using the following formula: [(nucleotide match-length)*(nucleotide identity/100)]/(nucleotide length of the candidate sRNA). A hierarchical clustering from the conservation score of candidate sRNAs was performed using Cluster 3.0 software [47] with centroid linkage. The conservation graph was generated using Java TreeView 1.1.5 [48].

### Deletion mutagenesis of sRNA-encoding genes

*E. amylovora* chromosomal deletion mutants were constructed using the red recombinase method [49]. Briefly, recombination fragments consisting of 50-nucleotide homology arms of flanking regions of sRNA encoding genes flanking a chloramphenicol resistance cassette were amplified from the plasmid pKD4. PCR products were purified by PCR purification and electroporated into *E. amylovora* Ea1189 expressing recombinase genes from the helper plasmid pKD46. Mutants were selected on LB medium amended with chloramphenicol. Deletion of target genes was confirmed by PCR and sequencing. Recombinant DNA work was approved by the Michigan State University Institutional Biosafety Committee (Registration #3122).

### Virulence assay

The virulence of wild type *E. amylovora* Ea1189 and mutant strains was tested using an immature pear fruit assay as previously described [8]. Briefly, for the immature pear fruit assay, bacteria were inoculated on wounded immature pears at a concentration of 1 × 10^4^ CFU ml^-1^, and the pears were incubated at 25°C under high relative humidity conditions. Lesion diameters were measured at 6 days post-inoculation. All assays were repeated three times, with five biological replicates in each experiment.

### Amylovoran production, swimming motility, and hypersensitive response (HR) assays

The amylovoran concentration in supernatants of *E. amylovora* cultures was quantified using a turbidity assay with cetylpyrimidinium chloride (CPC) [50]. Cells from overnight LB cultures were harvested by centrifugation, washed with phosphate-buffered saline (PBS), and inoculated into MBMA medium supplemented with 1% sorbitol. The supernatant of the MBMA culture was collected at 36 hrs post-inoculation following centrifugation of the culture. The amylovoran concentration in the supernatant was determined by adding 50 μl of CPC (50 mg ml^-1^) per ml of supernatant sample, followed by measuring the optical density OD_600_. The experiments were repeated three times with four biological replicates in each experiment.

To measure bacterial swimming motility, cells were cultured on LB agar plates for 48 hr. Cells were inoculated from the LB agar plates onto the center of swarming agar plates (10 g tryptone, 5 g NaCl, 2.5 g agar per liter) using an inoculation needle. Swimming diameters were measured at 20 hr post-inoculation. The experiments were repeated three times with four biological replicates in each experiment. For the HR assay, strains were cultured in LB broth overnight, harvested by centrifugation and washed with 0.5 × PBS twice. Cells were resuspended in 0.5 × PBS and adjusted to a concentration of 5 × 10^7^ CFU ml^-1^. Approximately 100 μl of cell suspension was infiltrated into 9 week-old *Nicotiana benthamiana* leaves using a needle-less syringe. The HR was observed at 16 hrs after infiltration.

### Biofilm quantification and analysis using scanning electron microscopy

To quantify the amount of biofilm formation using crystal violet staining, bacterial strains were cultured in 0.5X LB broth at 28°C in a 24-well plate with a glass cover slip placed in each well at a 30° angle. After 48 hr incubation, the bacterial culture was removed from the wells and 10% crystal violet was added into the wells. After incubation at 28°C for 1 hr, the glass cover slips were rinsed with water, air dried for 4 hours, and eluted with 200 μl of elution solution (40% methanol, 10% glacial acetic acid). The solubilized crystal violet in the elusion solution was quantified by measuring the OD_600_ absorbance using a Safire microplate reader (Tecan; Research Triangle Park, NC). The experiment was repeated three times with 12 replicates in each experiment.

The observation of biofilm formation using scanning electron microscopy (SEM) was performed as described previously [39]. Briefly, strains were cultured in 0.5X LB broth in a 96 well plate with a 300 mesh TEM gold grid in each well (G300-Au, Electron Microscopy Sciences; Hatfield, PA). After incubation at 28°C for 48 hr, 100 μl of paraformaldehyde-gluteraldehyde (2.5% of each compound in 0.1 M sodium cacodylate buffer, Electron Microscopy Sciences) was added to each well. The mixture was incubated at 25°C for 1 hr, and grids were dehydrated successively in 25, 50, 75, and 90% ethanol for 30 min each and in 100% ethanol three times for 15 min. Grids were then critical point dried using a critical point drier (Balzers CPD; Lichtenstein) and mounted on aluminum mounting stubs (Electron Microscopy Sciences). Samples were then coated with osmium using a pure osmium coater (Neoc-an; Meiwa Shoji Co. Ltd., Japan). Images were taken on a JEOL 6400 V scanning electron microscope (Japan Electron Optics Laboratories) equipped with an LaB6 emitter (Noran EDS) using analySIS software (Soft Imaging System; GmbH).

## Results

### Identification of Hfq-dependent sRNAs by RNA-seq

To identify Hfq-dependent sRNAs, Illumina deep sequencing (RNA-seq) was performed to identify small intergenic RNA transcripts whose expression was reduced in the absence of *hfq*. Wild type *E. amylovora* Ea1189 and the deletion mutant Ea1189Δ*hfq* were cultured for 6 and 12 hr in Hrp-inducing minimal medium, conditions that induce the expression of T3SS and other virulence genes [5]. Total bacterial RNA was isolated from Ea1189 6 hr, Ea1189 12 hr, Ea1189Δ*hfq* 6 hr and Ea1189Δ*hfq* 12 hr. Small RNAs ranging from 50 - 350 nt were enriched from the total RNAs and sequenced by the Illumina HiSeq 2000 system. A total of 213 million 50-nt paired reads were obtained. Of these reads, a total of 199 million reads passed quality control and were used for mapping to the genome of *E. amylovora* ATCC 49964, and 148 million reads were successfully mapped. From these, 78 million were excluded as alignments showed that they mapped to already annotated rRNA or tRNA genes. The remaining reads (Ea1189 6 hr, 22 million; Ea1189 12 hr, 9 million; Ea1189Δ*hfq* 6 hr, 32 million; Ea1189Δ*hfq* 12 hr, 7 million) were used for the identification of sRNAs in the intergenic regions in the genome of *E. amylovora*.

**Figure 1:**
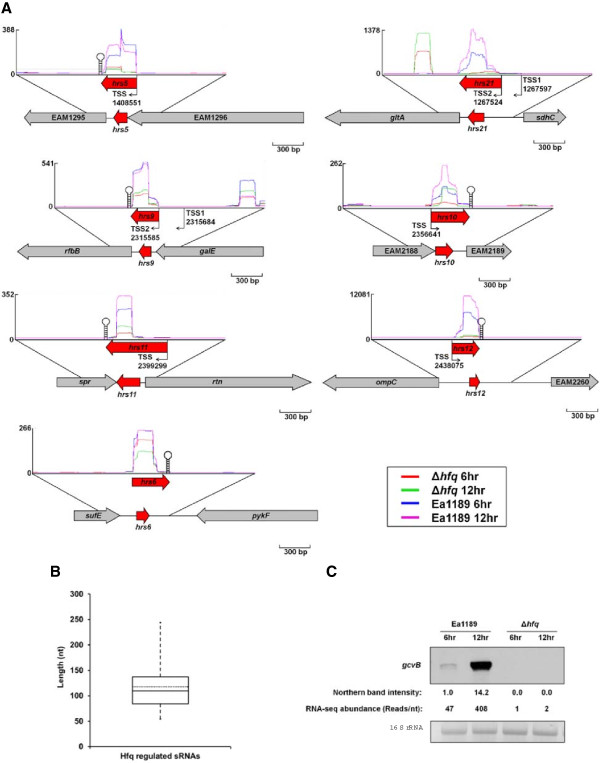
**Identification of sRNAs using RNA-seq transcriptomics. (A)** Illustration of examples of sRNAs identified by RNA-seq. **(B)** sRNA length distribution. The box and whisker plot diagram represents the minimum and maximum size, the median as well as the average sizes of the sRNA identified. **(C)** Comparison of the GcvB RNA amount detected by Northern blot and by RNA-seq.

We searched for small transcripts that aligned to intergenic regions of the *E. amylovora* genome, with significantly reduced expression in Ea1189Δ*hfq* 6 hr and Ea1189Δ*hfq* 12 hr compared to Ea1189 6 hr and Ea1189 12 hr, respectively. The candidate intergenic transcripts were further inspected manually to exclude transcripts that were extensions from the adjacent ORFs. Transcripts that contain ORFs, riboswitches, and transcriptional regulatory structures such as Jumpstart structures and Phe leaders were also excluded. The remaining intergenic transcripts were annotated as candidate Hfq-dependent sRNAs. A total of 38 candidate Hfq-dependent sRNAs were identified (Table 1, Figure 1A). These sRNAs ranged from 54 to 244 nt with a median size of 110 nt and average size of 118 nt (Figure 1B).

**Table 1:** **sRNA-encoding genes in** ***Erwinia amylovora*** **identified by RNA-seq**

ID^a^	Strand^b^	Start^c^	End^c^	Size^d^	Adjacent genes^e^	Orientation^f^	Rfam (E-value)^g^	RI terminator^h^	Northern^i^	Mutation^j^	5’ RACE^k^	Δ ***hfq*** /Ea1189 6 hr (%)^l^	Δ ***hfq*** /Ea1189 12 hr (%)^m^
*spf* (*spot42*)	+	52654	52816	163	*polA/engB*	> > <	RF00021 (3.2E-25)	Yes	NT	Yes		12.5	7.2
*hrs1*	+	130070	130143	74	*cpxP*/*fief*	> > >	N/A	Yes	Yes	No		21.2	13.9
*hrs2*	+	245012	245077	66	*metE*/*ysgA*	> > <	N/A	Yes	NT	No		8.5	4.5
*hrs3*	+	834512	834619	108	EAM0472/EAM0473	< > <	N/A	Yes	NT	No		33.3	0.0
*micM* (*sroB*)	+	1149210	1149296	87	EAM1042/EAM1043	< > <	RF00368 (7.5E-10)	Yes	NT	Yes		10.0	0.0
*hrs4*	-	1252343	1252249	95	*mtr*/*fur*	> < <		Yes	NT	No		86.9	76.4
*hrs5*	-	1408551	1408436	116	EAM1295/EAM1296	< < <	RF00110 (1.2E-10)	Yes	Yes	Yes	1408551	15.9	10.8
*rprA*	-	1771945	1771835	111	*ppsA*/EAM1647	> < <	RF00034 (1.9E-12)	Yes	Yes	Yes		2.5	5.5
*hrs6* (*rmaA*)	+	1783452	1783564	113	*sufE/lpp*	> > <	N/A	Yes	Yes	Yes		76.7	50.4
*hrs7*	-	1964168	1964063	78	*palI/*EAM1824	< < <	N/A	Yes	NT	No		8.7	0.0
*ryhB*	-	1981794	1981655	140	*pspF/sapA*	> < >	RF00057 (6.9E-13)	Yes	Yes	Yes		2.7	2.1
*hrs8*	-	2132995	2132831	165	EAM1984/EAM1985	< < <	RF00111 (1.1E-24)	Yes	Yes	Yes	2132931	24.2	15.2
*hrs9*	-	2315585	2315491	95	EAM2160/*galE*	< < <	RF01707 (2.5E-11)	Yes	Yes	No	2315684; 2315585	29.6	39.2
*hrs10*	+	2356621	2356768	148	EAM2188/EAM2189	> > >	N/A	Yes	Yes	Yes	2356641	24.0	42.1
*hrs11*	-	2399219	2399091	129	*spr/rtn*	> < >	N/A	Yes	ND	Yes	2399299	18.6	29.2
*hrs12*	+	2438083	2438169	87	*ompC/*EAM2260	< > >	RF00033 (6.3E-3)	Yes	Yes	Yes	2438075	3.5	5.2
*hrs13*	-	2610835	2610754	82	*ansP*/EAM2411	< < <	N/A	Yes	Yes	Yes		0.3	11.2
*micA*	+	2872049	2872162	114	*luxS/gshA*	< > <	RF00078 (5.6E-13)	Yes	NT	Yes		68.5	26.3
*gcvB*	+	2962739	2962947	209	*gcvA/*EAM2720	< > <	RF00022 (3.4E-33)	Yes	Yes	No		2.1	0.5
*omrAB*	-	3009426	3009347	80	EAM2752/EAM2753	< < >	RF00079 (1.4E-14)	Yes	Yes	Yes		28.0	4.8
*arcZ* (*ryhA*)	+	3399347	3399550	204	*mtgA/arcB*	< > <	RF00081 (8.4E-16)	Yes	Yes	Yes		3.1	1.6
*hrs15*	-	3573610	3573476	135	EAM3277/EAM3278	< < >	RF00057 (5.0E-13)	Yes	NT	Yes		5.9	6.3
*hrs16*	+	3790560	3790675	116	EAM3469/EAM3470	> > >	N/A	Yes	NT	No		0.0	0.0
*hrs17*	+	83003	83246	244	EAM0051/EAM0052	< > >	N/A	No	NT	No		31.1	18.2
*hrs18*	+	500345	500429	85	*dcuA/aspA*	< > <	N/A	No	NT	No		51.0	36.5
*hrs19*	+	1119874	1119949	76	EAM1011/EAM1012	< > <	N/A	No	Yes	No		57.4	73.3
*hrs20*	-	1212602	1212522	81	EAM1116/EAM1117	> < >	N/A	No	NT	Yes		16.5	42.9
*hrs21*	-	1267525	1267392	134	*gltA/sdhC*	< < >	N/A	No	Yes	Yes	1267597; 1267524	10.9	2.2
*hrs23*	+	1767030	1767167	138	*hmuS/*EAM1643	< > <	N/A	No	NT	No		38.1	62.1
*hrs24*	-	1794862	1794772	91	EAM1664/EAM1665	< < >	N/A	No	NT	No		2.4	0.0
*hrs25*	-	1894012	1893924	89	EAM1754/EAM1755	< < >	N/A	No	NT	No		24.1	11.8
*hrs26*	-	1909866	1909756	111	*pepT*/EAM1768	> < <	N/A	No	NT	No		11.1	3.3
*hrs27*	+	1922305	1922405	101	EAM1774/*fnr*	< > >	N/A	No	Yes	Yes		0.0	0.0
*hrs28*	-	1929971	1929863	109	EAM1781/*ydgI*	> < >	N/A	No	NT	No		5.0	3.4
*hrs29*	+	2391802	2392008	207	*nfo/fruA*	> > <	N/A	No	NT	Yes		13.3	28.6
*hrs30*	+	2493229	2493312	84	EAM2298/EAM2299	< > <	N/A	No	NT	No		59.1	44.6
*hrs31*	-	2556693	2556613	81	*ccmA/*EAM2395	< < >	N/A	No	Yes	Yes		65.2	3.5
*hrs32*	+	2649467	2649520	54	EAM2427/EAM2428	< > <	N/A	No	NT	No		40.0	69.2
*hrs33*	-	2857326	2857206	121	EAM2616/EAM2617	> < >	N/A	No	NT	No		58.5	50.0
*hrs34*	-	3356002	3355930	73	EAM3063/EAM3064	< < >	N/A	No	NT	Yes		18.2	46.7

^a^Gene names of *E. amylovora* sRNAs.

^b^The strand (+:clockwise; -:counterclockwise strand of the chromosome) that the sRNA is encoded.

^c^Genome locations of the sRNA genes*.*

^d^Putative sizes of the sRNAs detected by deep sequencing.

^e^Flanking genes of the intergenic region in which the sRNA is encoded.

^f^The orientation of the flanking genes and sRNA gene (middle).

^g^The Rfam database match. Rfam accession number and E-value are provided if a match was found. N/A means no match found in the Rfam database.

^h^Whether Rho-independent terminator sequences are present at the 3’ end of the sRNA.

^i^Whether the expression and size of the sRNA were confirmed by Northern blot. See Figure 2 and [39]. ND: not detected, NT: not tested.

^j^Whether deletion mutant of the sRNA gene was constructed.

^k^Whether the transcription start site of the sRNA was mapped by the 5’ RACE assay. The number indicates the nucleotide on the *E. amylovora* genome from which the transcription of the sRNA starts.

^l^The percentage of sRNA depth in Δ*hfq* mutant compared to Ea1189 at 6 hr post-induction in Hrp-inducing MM.

^m^The percentage of sRNA depth in Δ*hfq* mutant compared to Ea1189 at 12 hr post-induction in Hrp-inducing MM.

Among all 38 putative sRNAs, 8 sRNAs (Spf, MicM, RprA, RyhB, MicA, GcvB, OmrAB, ArcZ) were identified in *E. amylovora* from a previous computational search based on sequence homology with known *E. coli* sRNAs [39]. We designated the other 30 sRNAs identified from this study as Hrs (*H*fq-*r*egulated *s*mall) RNAs. To determine if any of these sRNAs were novel sRNAs, sequences of the 30 sRNAs were compared against the Rfam database [51]. Orthologs of four *E. amylovora* sRNAs from this study (Hrs5, Hrs8, Hrs12 and Hrs15) were identified in the Rfam database (RybB, RyeB, MicF and RyhB, respectively, Table 1). Of note, the sequences of RyhB (140 nt), identified previously, and Hrs15 (135 nt) were aligned and shown to be 73% identical (data not shown). The remaining 26 sRNAs are novel sRNAs identified for the first time. Rho-independent terminator sequences were also searched at the 3’ end of the sRNAs, and 21 of the 38 sRNAs contained typical Rho-independent terminator sequences with GC-rich stem-loop structure and oligo-Us whereas the remaining 17 sRNAs did not contain typical Rho-independent terminators.

The abundance of sRNAs in the pools from Ea1189 6 hr, Ea1189 12 hr, Ea1189Δ*hfq* 6 hr and Ea1189Δ*hfq* 12 hr was quantified based on the reads of deep sequencing using Artemis (Table 2). All sRNAs identified showed significant reductions in abundance in Ea1189Δ*hfq* compared with Ea1189 at both 6 hr and 12 hr (Table 2, Figure 1A). To validate the accuracy of the RNA abundance determined by RNA-seq, the RNA amount of GcvB in Ea1189 6 hr, Ea1189 12 hr, Ea1189Δ*hfq* 6 hr, and Ea1189Δ*hfq* 12 hr was measured by Northern blot analysis (Figure 1C). Consistent with the RNA-seq result, GcvB was only detected in Ea1189 but not in Ea1189Δ*hfq* at both 6 hr and 12 hr after induction (Figure 1C). In addition, a 14.2-fold increase of GcvB RNA was detected in Ea1189 12 hr compared to its amount at 6 hr (Figure 1C), which is similar to an 8.7-fold induction (9823 reads in Ea1189 6 hr compared to 85272 reads in Ea1189 12 hr, Table 2) detected by RNA-seq. These results indicate that the quantification of sRNAs by Illumina deep sequencing in this experiment is accurate and reliable.

**Table 2:** **The abundance of sRNAs in** ***E. amylovora*** **Ea1189 detected by RNA-seq**

ID	Ea1189 6 hr	Ea1189 12 hr	Δ***hfq*** 6 hr	Δ***hfq*** 12 hr	Δ ***hfq/*** Ea1189 6 hr (%)	Δ ***hfq/*** Ea1189 12 hr (%)
Hrs12	407073^a^	681297	14094	35583	3.5	5.2
ArcZ (RyhA)	103836	201144	3264	3264	3.1	1.6
Spf (Spot42)	82478	153057	10269	11084	12.5	7.2
Hrs1	80364	20202	17020	2812	21.2	13.9
RprA	58053	87135	1443	4773	2.5	5.5
Hrs4	52345	15295	45505	11685	86.9	76.4
Hrs13	52070	71176	164	7954	0.3	11.2
Hrs31	50706	53460	33048	1863	65.2	3.5
Hrs21	41942	83884	4556	1876	10.9	2.2
Hrs9	29165	27835	8645	10925	29.6	39.2
Hrs5	21112	22620	3364	2436	15.9	10.8
Hrs19	20520	6840	11780	5016	57.4	73.3
Hrs20	19683	26082	3240	11178	16.5	42.9
Hrs11	15222	22059	2838	6450	18.6	29.2
Hrs2	13992	10362	1188	462	8.5	4.5
Hrs6 (RmaA)	13560	13447	10396	6780	76.7	50.4
Hrs17	10980	10736	3416	1952	31.1	18.2
Hrs8	10230	31515	2475	4785	24.2	15.2
GcvB	9823	85272	209	418	2.1	0.5
Hrs10	7400	14060	1776	5920	24.0	42.1
Hrs27	6363	6868	0	0	0.0	0.0
Hrs23	5796	34224	2208	21252	38.1	62.1
RyhB	5180	13580	140	280	2.7	2.1
MicA	4332	11286	2850	2964	65.8	26.3
Hrs18	4165	4420	2125	1615	51.0	36.5
OmrAB	4000	3360	1120	160	28.0	4.8
Hrs24	3822	1001	91	0	2.4	0.0
Hrs29	3105	1449	414	414	13.3	28.6
Hrs34	2409	1095	438	511	18.2	46.7
Hrs15	2295	2160	135	135	5.9	6.3
Hrs28	2180	6431	109	218	5.0	3.4
Hrs33	2057	6292	1210	3146	58.8	50.0
Hrs30	1848	4704	1092	2100	59.1	44.6
Hrs7	1794	1638	156	0	8.7	0.0
Hrs32	1350	6318	540	4374	40.0	69.2
Hrs26	999	666	111	22	11.1	3.3
MicM (SroB)	870	435	87	0	10.0	0.0
Hrs25	739	757	178	89	24.1	11.8
Hrs16	696	348	0	0	0.0	0.0
Hrs3	324	324	108	0	33.3	0.0

^a^The abundance (per base depth) = the total number of reads aligned to the sRNA gene/the length of the sRNA gene.

### Identification of Hfq-dependent sRNAs by Rho-independent terminator search

Rho-independent terminators are often considered as landmarks for the computational identification of Hfq-dependent sRNAs in bacterial genomes [52]. We performed an independent search for Hfq-dependent sRNAs by first mapping all the Rho-independent terminators located within intergenic regions, and then identifying sRNAs by detecting short-length transcriptional activity upstream of any of these terminators. To map the Rho-independent terminators, first, we compared sequences of the last 35 nucleotides (Rho-independent terminator sequences) of eight confirmed sRNAs in *E.amylovora* (Spf, MicM, RprA, RyhB, MicA, GcvB, OmrAB, ArcZ). Sequence alignment did not reveal any sequence conservation of the Rho-independent terminators of the eight sRNAs (data not shown). However, some common characteristics were observed among all the terminator sequences: containing at least 6 oligo-Us at the 3’ end; containing at least 4 G + Cs in the last 6 nucleotides immediately upstream of the oligo-Us; containing at least 50% G + Cs in the last 25 nucleotides immediately upstream of the oligo-Us; and containing stem-loop RNA secondary structures in the GC-rich sequences upstream of oligo-Us.

With these characteristics, we performed a genome-wide search for putative Rho-independent terminators in the genome of *E. amylovora* ATCC 49964. Using bioinformatics approaches, 117 putative Rho-independent terminators were identified (Additional file 1: Table S2). Next, we examined if transcriptional activity was present upstream of these Rho-independent terminators using the RNA-seq data. The majority (60%) of these putative terminators showed transcriptional activity immediately upstream which stopped at the terminator sequences, suggesting that these sequences are actual Rho-independent terminators (Additional file 1: Table S2). A total of 23 Rho-independent terminators identified in this search showed transcriptional activity within 300 nt immediately upstream of the terminators in the intergenic regions, with reduced abundance in Ea1189Δ*hfq* 6 hr and 12 hr compared to Ea1189 6 hr and 12 hr (Additional file 1: Table S2). These 23 transcripts were selected as potential Hfq-dependent sRNAs.

We compared the 23 sRNAs identified in this search with the 38 sRNAs identified from the RNA-seq experiment. Our results showed that 21 of the 23 Hfq-dependent sRNAs identified in the terminator search were also identified in the deep sequencing search. Two sRNAs (Hrs3 and Hrs16) were not identified in the RNA-seq search, because of low RNA abundance. These results suggest that the bioinformatics prediction of Rho-independent terminators is a good complementary guideline for the identification of Hfq-dependent sRNAs. The fact that most sRNAs identified in the Rho-independent terminator search were also identified in the deep sequencing search also indicates that the identification of sRNAs by deep sequencing is accurate. In combination with RNA-seq and Rho-independent terminator searches, a total number of 40 candidate sRNAs (38 from RNA-seq search and 2 from the Rho-independent terminator search) were identified (Table 1).

### Validation of the expression and sizes of sRNAs by Northern blot

**Figure 2:**
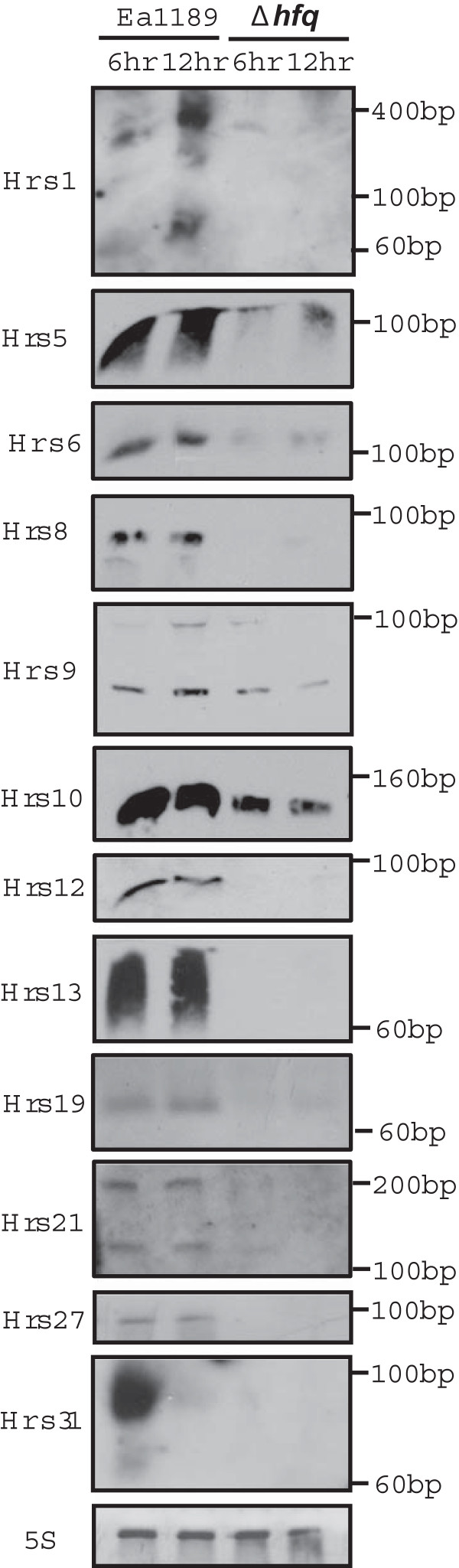
**Northern blot detection of the sRNAs in Ea1189 and Ea1189Δ** ***hfq*** **at 6 and 12 hrs post-inoculation in Hrp-inducing minimal medium.** 5S rRNA was used as the loading control. A biotin-labeled RNA marker was used to estimate the sizes of the sRNAs. sRNA Hrs5, Hrs6, Hrs8, Hrs10, Hrs12, Hrs13, Hrs19, Hrs27 have only one major band whereas sRNA Hrs1, Hrs9, Hrs21, and Hrs31 have two major bands.

To validate the expression and confirm the size of the sRNAs identified by RNA-seq, 13 novel sRNAs, including 9 sRNAs with and 4 sRNAs without Rho-independent terminators, were analyzed by Northern blot. The expression of 12 sRNAs was detected (Figure 2), and one sRNA, Hrs11, was not detected (data not shown). In addition, consistent with deep sequencing observations, significantly-reduced expression of most sRNAs was observed in Ea1189Δ*hfq* compared with Ea1189 (Figure 2). Our results also showed that the sizes of sRNAs determined by Northern blot are approximately the same as those determined by RNA-seq, with one exception being the sRNA Hrs8, whose major transcript detected by Northern blot is smaller than the size determined by RNA-seq (Table 1 and Figure 2).

### Validation of the transcriptional start sites of sRNAs by 5’RACE

Next, we performed a 5’ RACE assay to validate the transcriptional start sites of 8 sRNA-encoding genes determined by RNA-seq, and the transcriptional start sites of 7 sRNAs were successfully mapped (Table 1). Of these, 5 sRNAs (Hrs5, Hrs8, Hrs10, Hrs11, and Hrs12) only showed one major transcriptional start site whereas two sRNAs (Hrs9 and Hrs21) showed two transcriptional start sites (Table 1). Similar transcriptional start sites (within 10 bp) of Hrs5, Hrs9, Hrs12 and Hrs21 were determined by 5’ RACE assay compared to the ones determined by deep sequencing assay. The transcriptional start sites of other sRNAs (Hrs8, Hrs10, and Hrs11) determined by 5’ RACE were 20-80 nt upstream of the sites determined by RNA-seq.

### Sequence conservation of the Hfq-dependent sRNAs

**Figure 3:**
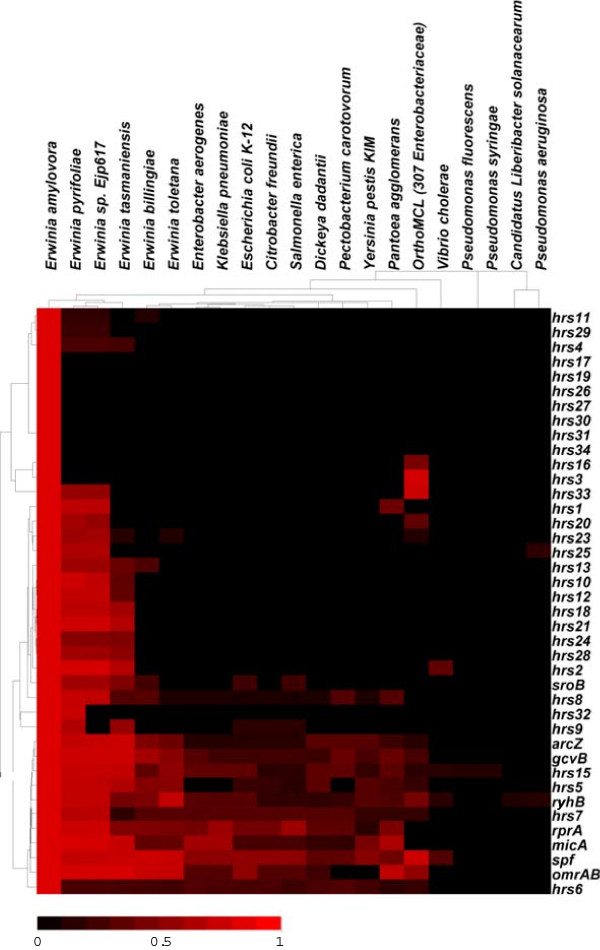
**The nucleotide sequence conservation of the 40** ***E. amylovora*** **Hfq-regulated sRNAs among 21 bacterial species.** Red indicates high nucleotide conservation and black indicates low conservation. Clustering of the sRNAs and bacterial species was done using Cluster 3.0 with centroid linkage.

To determine the sequence conservation of the Hfq-dependent sRNAs identified in *E. amylovora*, 20 bacterial genomes from four families of Gamma Proteobacteria, including 15 Enterobacteriaceae species, three Pseudomonadaceae species, one Vibrionaceae species and one Rhizobiaceae species, were searched for orthologs of the sRNAs identified in this study. The nucleotide conservation scores were calculated by Blast search, clustered, and depicted by Java tree view. Based on the sequence conservation, the 40 sRNAs identified in *E. amylovora* could be clustered into three groups (Figure 3). Group 1, including 11 sRNAs (ArcZ, GcvB, Hrs15, Hrs5, RyhB, Hrs7, RprA, MicA, Spf, OmrAB and Hrs6), is conserved among most Enterobacteriaceae species examined. Among them, 7 sRNAs (ArcZ, GcvB, Hrs15, RyhB, Hrs7, Spf, and Hrs6), were conserved in all Enterobacteriaceae. Group 2, including 16 sRNAs (Hrs1, Hrs20, Hrs23, Hrs25, Hrs13, Hrs10, Hrs12, Hrs18, Hrs21, Hrs24, Hrs28, Hrs2, SroB, Hrs8, Hrs32, and Hrs9), is conserved in the *Erwinia* genus, but not in other *Enterobacteriaceae* species. All 16 sRNAs in group 2 are conserved in *E. amylovora* and the related plant pathogen *E. pyrifoliae*, and 14 of them are conserved in *E. amylovora*, *E. pyrifoliae*, and another related pathogen *Erwinia* sp. Ejp617 [53] (Figure 3). Eleven and three group 2 sRNAs are also conserved in the non-pathogenic plant epiphytes *E. tasmaniensis* and *E. billingiae*, respectively. Group 3, including the other 13 sRNAs, is mostly only conserved in *E. amylovora* (Figure 3).

### sRNA expression profile at 6 and 12 hour of induction in Hrp-inducing minimal medium

**Figure 4:**
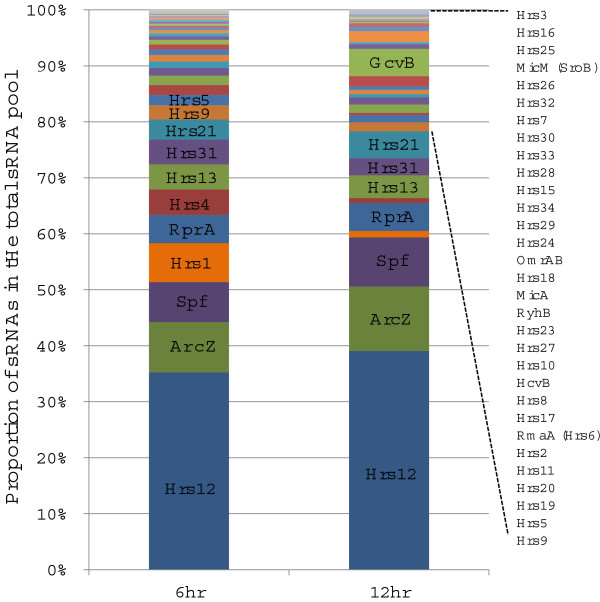
**The expression profile of Hfq-regulated sRNAs in Ea1189 at 6 and 12 hrs of post-inoculation in Hrp-inducing minimal medium.** The percentage of the reads of each individual sRNA in comparison to the total sRNA reads is depicted by bars with various colors.

Next, we determined how the expression of sRNAs changed following incubation of test strains in Hrp-inducing minimal medium, a medium that mimics the plant environment and induces the expression of T3SS and other virulence genes [5]. A sRNA distribution graph was generated by calculating and displaying the percentage of reads of each individual sRNA against the total reads of all sRNAs in Ea1189, at 6 and 12 hours post-inoculation (Figure 4 and Additional file 1: Table S3). Total sRNA reads increased from 1381341 at 6 hr to 1908049 at 12 hr. The most abundant sRNA at both time points was Hrs12 (29.5% of the total at 6 hr and 35.7% at 12 hr), and the least abundant sRNA is Hrs3 (0.02% at 6 hr and 0.02% at 12 hr). The 12 most abundant sRNAs comprised more than 80% of the total sRNAs. Comparing to the early induction at 6 hrs, the proportion of each sRNA in the total sRNAs at late stage of induction at 12 hrs also changed. Among the most abundant 12 sRNAs, 6 sRNAs (GcvB, Hrs21, AcrZ, Spf, Hrs12, and RprA) showed increased expression (6.3, 1.5, 1.4, 1.3, 1.2, and 1.1 fold, 12 hr/6 hr, respectively). The other 4 sRNAs (Hrs13, Hrs31, Hrs4, and Hrs1) showed decreased expression (1.0, 0.8, 0.7, 0.2, 0.2, and 0.1 fold decrease, 12 hr/6 hr, respectively).

### Virulence of 15 sRNA mutants

**Figure 5:**
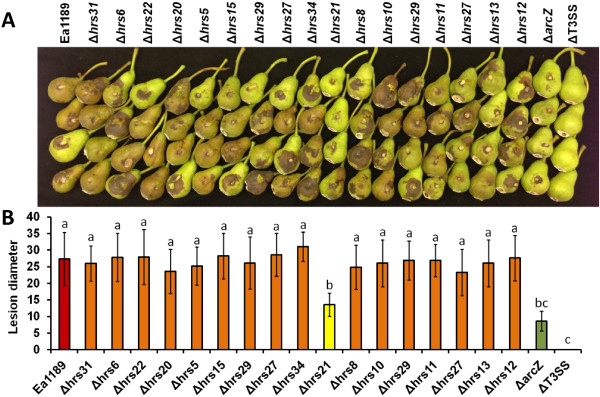
**Virulence of** ***E. amylovora*** **and the sRNA deletion mutants. (A)** Virulence of Ea1189, Ea1189ΔT3SS, and sRNA deletion mutants in immature pears at 5 day post-inoculation. **(B)** Average lesion diameters of immature pears inoculated with Ea1189, Ea1189ΔT3SS and the indicated sRNA deletion mutants of Ea1189. Sample means were compared by an analysis of variance and separated using the Student *t* test. The presence of different letters indicates that the means were significantly different (*P<*0.05).

In our previous report, deletion of the *hfq* gene led to significantly reduced virulence in *E. amylovora*[39]. To test if any Hfq-dependent sRNAs contribute to the virulence regulation, we constructed deletion mutants of 15 small RNAs identified in this study (Additional file 1: Table S1). The virulence of the sRNA mutants was compared with Ea1189 using an immature pear fruit assay. Two mutants, Ea1189ΔT3SS and Ea1189Δ*arcZ*, a sRNA mutant that showed significantly reduced virulence in our previous study [39], were also included as controls. At 6 days post-inoculation, necrosis and oozing were observed on pears inoculated with the wild type Ea1189, whereas no disease symptoms and reduced disease symptoms were observed on pears inoculated with Ea1189ΔT3SS and Ea1189Δ*arcZ*, respectively. Compared to the wild type Ea1189, most sRNA mutants did not show any significant difference in virulence (Figure 5). However, the virulence of one sRNA mutant, Ea1189Δ*hrs21*, was significantly attenuated compared with Ea1189.

### Hrs6, OmrAB, and ArcZ positively control bacterial motility

**Figure 6:**
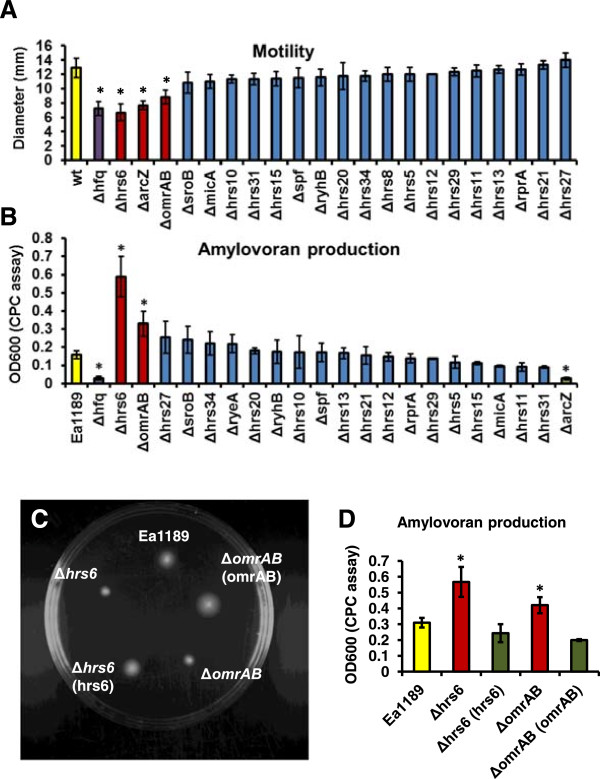
**Swimming motility and amylovoran production of wild type Ea1189 and the deletion mutants of sRNAs in Ea1189. (A)** Motility (diameters measured at 17 hrs post-inoculation). **(B)** Amylovoran production measured at 36 hrs post-inoculation in MBMA medium, by CPC assay. **(C)** Swimming motility of Ea1189, Ea1189Δ*omrAB*, Ea1189Δ*hrs6*, as well as Ea1189Δ*omrAB* and Ea1189Δ*hrs6* carrying complementation plasmids pMLomrAB and pMLhrs6. **(D)** Amylovoran production of Ea1189, Ea1189Δ*omrAB*, Ea1189Δ*hrs6*, as well as Ea1189Δ*omrAB* and Ea1189Δ*hrs6* carrying complementation plasmids pMLomrAB and pMLhrs6. Asterisks indicate significant difference (*P* < 0.05) compared to Ea1189.

The sRNA chaperone Hfq positively controls motility in *E. amylovora*[39]. To investigate if this positive regulation is through any Hfq-dependent sRNAs, the swimming motility of 21 sRNA mutants was tested on soft agar plates (Figure 6A). The wild-type Ea1189 was motile on the soft agar plate within 17 hr post-inoculation, whereas the motility of Ea1189Δ*hfq* was greatly reduced compared to Ea1189. Similar to Ea1189Δ*hfq*, the motility of three sRNA mutants, Ea1189Δ*hrs6*, Ea1189Δ*omrAB*, and Ea1189Δ*arcZ*, was significantly reduced. The reduced motility was able to be complemented (Figure 6C, the complementation of Ea1189Δ*arcZ* was reported in a different manuscript [39]. This suggests that Hrs6, OmrAB, and ArcZ positively control motility in *E. amylovora* in conjunction with the RNA chaperone Hfq.

### ArcZ positively controls amylovoran production, whereas Hrs6 and OmrAB negatively control amylovoran production

The sRNA chaperone Hfq positively regulates amylovoran production in *E. amylovora*[39]. We screened 21 sRNA mutants for mutants with altered amylovoran production (Figure 6B). Similar to the reduction in amylovoran production in Ea1189Δ*hfq*, the amylovoran production of Ea1189Δ*arcZ* was also reduced compared to Ea1189. However, increased amylovoran production was observed in two sRNA mutants, Ea1189Δ*hrs6* and Ea1189Δ*omrAB*. The altered amylovoran production in Ea1189Δ*hrs6*, Ea1189Δ*omrAB*, and Ea1189Δ*arcZ* was able to be complemented (Figure 6D and data not shown). This suggests that ArcZ positively controls amylovoran production similar to the RNA chaperone Hfq, whereas Hrs6 and OmrAB negatively control amylovoran production in *E. amylovora*.

### ArcZ is an important regulator of the type III secretion system

**Figure 7:**
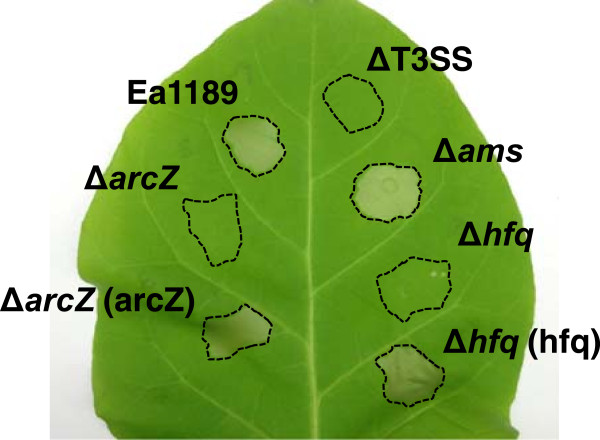
**Hypersensitive response (HR) elicited by** ***E. amylovora*** **Ea1189, Ea1189Δ** ***arcZ*** **, Ea1189Δ** ***hfq*** **, Ea1189ΔT3SS, Ea1189Δ** ***ams*** **, as well as Ea1189Δ** ***arcZ*** **and Ea1189Δ** ***hfq*** **carrying complementation plasmids pMLarcZ and pMLhfq.** Bacterial strains (1 × 10^7^ CFU ml^-1^) were infiltrated into *Nicotiana benthamiana* leaves and the HR was observed at 20 hr post-infiltration.

The Ea1189Δ*hfq* mutant failed to elicit a hypersensitive response (HR) when injected into leaves of the non-host *Nicotiana benthamiana*, suggesting that Hfq is required for the normal function of the type III secretion system [39]. To understand if the regulation of the T3SS by Hfq is through any of the sRNAs identified in this study, 21 mutants of Hfq-dependent sRNAs were tested for the HR causing ability. Similar to Ea1189Δ*hfq*, one sRNA mutant Ea1189Δ*arcZ* also showed a significantly-reduced HR (Figure 7). The reduced HR was able to be restored to the wild type level by complementation. This suggests that the sRNA ArcZ is required for the normal function of the T3SS, similar to its chaperone Hfq.

### ArcZ controls bacterial attachment and biofilm formation

**Figure 8:**
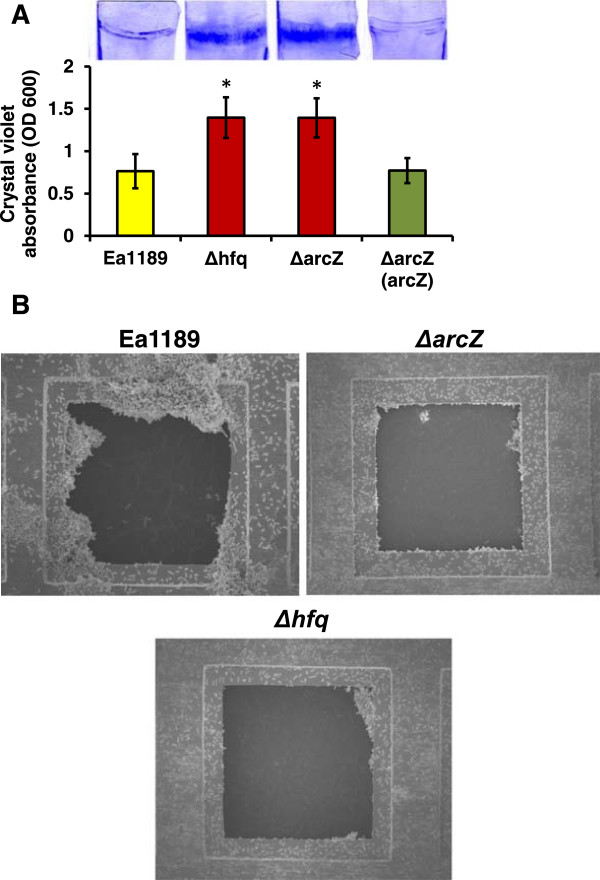
**Biofilm formation of Ea1189, Ea1189Δ** ***hfq*** **, and Ea1189Δ** ***arcZ*** **. (A)** Biofilm formation of Ea1189, Ea1189Δ*hfq*, Ea1189Δ*arcZ*, and Ea1189Δ*arcZ* carrying complementation plasmid pMLarcZ on glass cover slips. Bacterial strains were incubated with glass cover slips in static cultures of 0.5X LB broth. The biofilm formed on the cover slips was stained with crystal violet, and quantified by measuring light absorbance at OD600. Asterisks indicate significant difference (*P* < 0.05) compared to Ea1189. **(B)** Biofilm formation and cell attachment of Ea1189, Ea1189Δ*hfq*, and Ea1189Δ*arcZ* on gold grid observed by scanning electron microsope. Images were taken at X1100 magnification.

In our previous report, we demonstrated that Hfq controls attachment and biofilm formation in *E. amylovora*[39]. To identify sRNAs that control biofilm formation, the biofilm formation of the sRNA mutants was determined using a crystal violet staining assay. Similar to Ea1189Δ*hfq*, Ea1189Δ*arcZ* formed an increased amount of biofilm on glass cover slips compared to Ea1189 after 48 hr of incubation (Figure 8A). When examined using electron microscopy, mature biofilm formation was observed in Ea1189 (Figure 8B). However, the majority of cells of Ea1189Δ*arcZ* and Ea1189Δ*hfq* observed were solitary, non-aggregated cells attaching to the grid surface (Figure 8B). Fewer cell aggregates with less complex structures were observed in Ea1189Δ*arcZ* and Ea1189Δ*hfq* compared to Ea1189 (Figure 8B). This suggests that similar to Hfq, ArcZ also promotes aggregation while repressing attachment.

## Discussion

In this study, we identified 40 candidate Hfq-dependent sRNAs in the plant pathogen *E. amylovora* and further demonstrated that four of them regulated various virulence traits including motility, amylovoran EPS production, biofilm formation, and the T3SS. Although sRNAs have been increasingly recognized as pivotal regulators in bacteria, genome-wide identification of sRNAs has only been performed in a limited number of bacteria. In plant pathogens in particular, sRNA identification using deep sequencing methods has been reported in only three bacterial species prior to this study. In a transcriptome analysis of *Pseudomonas syringae*, transcription of 19 of the 21 non-coding RNAs predicted by Rfam database was detected [22], and three previously unannotated non-coding RNAs (*psr1*, *psr2*, and *psr3*) were also discovered. In *Xanthomonas campestris*, 23 sRNAs were identified from a genome-wide transcriptome analysis by deep sequencing, and one sRNA, sX12, was identified as a virulence regulator [21]. In *Agrobacterium tumefaciens*, 26 sRNAs were identified by combining a comparative bioinformatics approach and a deep sequencing approach [23, 54]. Compared to these studies which identified *trans-* and *cis-*encoded sRNAs, our work specifically focused on the identification of the *trans-* encoded sRNAs that are regulated by the RNA chaperone Hfq. The number of sRNAs identified in this study, 40, is comparable to the number of sRNAs identified in the bacterial species mentioned above and in closely related species such as *E.coli* (about 107 sRNAs in *E. coli* K-12, documented in the Rfam database).

Hfq-dependent sRNAs are a major group of bacterial sRNAs whose stability and function are dependent on the RNA chaperone Hfq. The deletion mutant of *hfq* in *E. amylovora* renders pleiotropic phenotypes including reduced motility and amylovoran production, increased attachment, disrupted T3 secretion and translocation, and reduced virulence [39]. This suggests that Hfq, as the global sRNA chaperone, may interact with multiple sRNAs that target various mRNAs to control different aspects of cellular and virulence processes. To test this hypothesis, we aimed to specifically identify the Hfq-dependent sRNAs and focus on their expression in Hrp-inducing minimal medium, a condition that mimics the *in planta* environment.

Two independent searches, based on RNA-seq and Rho-independent terminator prediction, were performed for the purpose of identifying Hfq-dependent sRNAs. RNA-seq identifies small, intergenic transcripts whose stabilities are dependent on Hfq. Although some sRNAs identified in the deep sequencing contain Rho-independent terminators, it was not clear whether the RNA-seq method had identified all sRNAs that possess Rho-independent terminators. To take the presence of Rho-independent terminator into consideration and to ensure that all the sRNAs with Rho-independent terminators are identified, we performed a second search by first mapping all of the Rho-independent terminators in the *E. amylovora* genome, and then identifying sRNA-encoding genes by detecting short-length transcriptional activity upstream of the terminators. The combination of the two searches identified Hfq-dependent sRNAs that possess Rho-independent terminators and sRNAs that do not contain Rho-independent terminators but depend on Hfq for their cellular stability.

Rho-independent terminators, which contain potential Hfq binding sequences, are considered to be important features of Hfq-dependent sRNAs [13, 15]. In this study, 17 of the sRNAs identified did not possess typical Rho-independent terminators although the abundance of these sRNAs was reduced in Ea1189Δ*hfq* compared to Ea1189. Prior to this work, sRNAs whose stabilities are dependent on the presence of Hfq but do not contain Rho-independent terminators have been observed in a few bacterial species. For example, 10 sRNAs were identified by RNA-seq in a study aiming to identify novel sRNAs in *E. coli*[24]. The abundance of five of them (*ychE-oppA*, *ytfL-msrA*, *glnA-typA*, *yhcF-yhcG*, and *yhcC-gltB*) showed significant reduction in an *hfq* mutant compared to the wild type *E. coli*. However, none of these five Hfq-dependent sRNAs possessed Rho-independent terminators [24]. In contrast, Rho-independent terminator sequences were identified in sRNAs whose stability is not dependent on Hfq, such as *ygfl-yggE*; and *ynfM-asr*. Similarly, in *Yersinia pseudotuberculosis*, some sRNAs whose abundance is Hfq dependent did not contain Rho-independent terminators, such as Ysr4 [28]. Our observation, along with previous observations, suggests the presence of sRNAs whose abundance is Hfq-dependent yet do not contain typical Rho-independent terminators in multiple species of the Enterobacteriaceae family. Further protein-RNA binding assays will elucidate whether Hfq directly interacts with these sRNAs or if the stabilization of the sRNAs by Hfq is indirect.

We observed a dynamic re-patterning of Hfq-dependent sRNAs between 6 and 12 hr induction in Hrp-inducing MM. In *E. amylovora*, the expression of key virulence genes is induced in Hrp-inducing minimal medium, and expression levels of some of these genes are at different levels between 6 and 12 hrs after inoculation. The change of proportion of an sRNA over time in this medium may indicate its role in modulation of virulence factors. Three of the sRNAs whose expression increased (AcrZ, RprA and Hrs21), are also virulence-regulating sRNAs. By increasing their expression in the Hrp-inducing medium, they may activate virulence-related genes at different timings or host locations during pathogenesis. In contrast, the relative abundance of Hrs6 and OmrAB dropped from 2.1% and 0.3% of the total sRNA pool at 6 hr post-induction to 1.5% and 0.2% at 12 hr post-induction, respectively. We also demonstrated that Hrs6 and OmrAB promote motility and limit amylovoran production (see Results). In *E. amylovora*, motility and amylovoran are two critical virulence determinants that are expressed at different stages of infection. Motility is believed to be critical for the early stage of infection, which enables *E. amylovora* to move from the stigma of the flower or at wound sites on leaves into the plants to establish infections. Biofilm formation is turned on at the later stage of infection to help *E. amylovora* to migrate into the xylem and cause systemic infections, and amylovoran is a critical component of biofilms formed by *E. amylovora*[4]. The fact that Hrs6 and OmrAB activate motility and repress amylovoran production, and that the abundance of Hrs6 and OmrAB dropped from 6 hr to 12 hr post-induction in Hrp-inducing minimal medium, suggest that *E. amylovora* may use sRNAs such as Hrs6 and OmrAB as a regulatory mechanism to transit from early to late stages of infection.

Besides the virulence-regulating sRNAs, the re-patterning of the expression of other sRNAs was also observed. The expression of GcvB increased the most from 6 hr to 12 hr (6.3-fold) among all sRNAs. A similar observation was made in *E. coli*, where GcvB was barely detectable at 3 hrs in M9 minimal medium, but was strongly expressed at 8 hrs induction when analyzed by Northern blot [55]. Likewise, the expression dynamics of Hrs5 in *E. amylovora* were similar to the expression of the ortholog RybB in *E. coli*[56]. Taken together, these observations suggest that the expression of conserved sRNAs in Enterobacteriaceae is similar across bacterial species, suggesting that some of the functions that these sRNAs possess are conserved among different species. The re-patterning of sRNA expression may also decide the regulatory activities of the sRNAs, since competitions of sRNAs for the availability of Hfq occurs, and more abundant sRNAs may have better access of Hfq and exert stronger regulation [57].

From this study and a previous study, we have identified four sRNAs (ArcZ, Hrs6, OmrAB, RprA) as virulence regulators in *E. amylovora*, and in some cases have identified the specific virulence determinants regulated. OmrAB, ArcZ and Hrs6 were identified as positive regulators of motility in this study. In contrast, OmrAB and ArcZ were shown to be negative regulators of motility and FlhDC, the master regulator of motility, in *E. coli*[58]. The over-expression of OmrAB and ArcZ led to reduced motility on soft agar plates, as well as reduced translation of *flhDC*[58]. This suggests that although OmrAB and ArcZ are motility regulators in both *E. amylovora* and *E. coli*, the regulatory mechanism may be different.

Hrs6 is a novel Hfq-dependent sRNA that was identified for the first time, and we demonstrated that Hrs6 inversely controls amylovoran production and motility in *E. amylovora*. Although not documented in the Rfam database, Hrs6 has high sequence conservation in many Enterobacteriaceae species (Figure 3). Since Hrs6 has not been previously characterized in other Enterobacteriaceae and in light of the functions identified in this study, here we name it RmaA (Regulator of *m*otility and *a*mylovoran A). The sequence and function of RmaA was documented in NCBI, with the accession number KJ372221. It would be interesting to further characterize the detailed regulatory mechanism of RmaA on motility and amylovoran production in *E. amylovora*, as well as the regulatory function of RmaA in other Enterobacteriaceae species.

ArcZ was identified as a virulence-regulating sRNA in our previous study [39], and we found in this study that ArcZ confers pleiotropic regulation on multiple virulence determinants including motility, amylovoran production, attachment, biofilm formation, and the type III secretion system. Our observations that the virulence regulation repertoire of ArcZ is very similar to that of the global sRNA chaperone Hfq suggests that ArcZ could be the most critical virulence regulating sRNA in *E. amylovora*. ArcZ was previously described as a positive regulator of the stationary sigma factor RpoS and a negative regulator of motility in *E. coli*[58, 59]. It is also known as a negative regulator of serine uptake, oxidative stress, and motility in *Salmonella*[60]. Additionally, ArcZ is characterized as one of the 34 sRNAs that are not required for murine virulence in *Salmonella enterica*[61]. To our knowledge, this is the first report describing the regulatory mechanism of ArcZ affecting virulence. This also suggests that a small RNA may play different regulatory roles in various pathogens.

## Conclusions

In summary, we used an experimental method and a computational method and successfully identified candidate Hfq-dependent sRNAs in the genome of *E. amylovora*. These results provide basis for the future characterization of the functions, evolution and conservation of these sRNAs in *E. amylovora*. In addition, multiple Hfq-dependent sRNAs were demonstrated to control various virulence functions. This observation, together with our previous finding that the RNA chaperone Hfq controls multiple virulence factors [39], suggests that the post-transcriptional regulation by Hfq and Hfq-dependent sRNAs may play an important role in virulence modulation in *E. amylovora*. We also characterized the motility and amylovoran regulation by a novel sRNA Hrs6 (renamed to RmaA), which is conserved in multiple Enterobacteriaceae species but not documented, for the first time. Finally, we demonstrated that ArcZ, which has a pleiotropic regulation of all major virulence factors characterized in *E. amylovora* so far, might be the most critical virulence-regulating sRNA in this pathogen.

## Authors’ information

QZ is a post-doctoral research associate in the Department of Plant, Soil, and Microbial Sciences at the Michigan State University. His research interest is in the post-transcriptional regulation of virulence in plant pathogenic bacteria using genomics and molecular biology tools as well as developing novel control methods of bacteria diseases using antisense RNA technology. GWS is a professor in the Department of Plant, Soil, and Microbial Sciences at the Michigan State University. His main research interests are in host-pathogen interactions of plant pathogenic bacteria and the regulation of pathogenesis. Additional long-term interests include devising sustainable strategies for controlling bacterial diseases of plants.

## Electronic supplementary material

Additional file 1: Table S1: Bacterial strains and plasmids used in this study and their relevant characteristics. [62, 63]. **Table S2.** Summary of the Rho-independent terminators identified in the genome of *E. amylovora*. **Table S3.** The percentage of sRNAs in the total sRNA pool in the wild type Ea1189 at 6 and 12 hrs post induction in Hrp-inducing minimal medium. (DOCX 37 KB)
