# Supplementary material for: Genome-wide identification of Hfq-regulated small RNAs in the fire blight pathogen Erwinia amylovora discovered small RNAs with virulence regulatory function
Source: BMC Genomics. 2014 May 31;15(1):414. doi: 10.1186/1471-2164-15-414 (PMC4070566; doi:10.1186/1471-2164-15-414)
Supplement: Supplementary file 1 — Additional file 1: Table S1: Bacterial strains and plasmids used in this study and their relevant characteristics. [62, 63]. Table S2. Summary of the Rho-independent terminators identified in the genome of E. amylovora. Table S3. The percentage of sRNAs in the total sRNA pool in the wild type Ea1189 at 6 and 12 hrs post induction in Hrp-inducing minimal medium. (DOCX 37 KB) [file 12864_2014_6148_MOESM1_ESM.docx]

TABLE S1 Bacterial strains and plasmids used in this study and their relevant characteristics

| **Strains and plasmids** | **Relevant characteristics^a^** | **Source or reference** |
| --- | --- | --- |
| *Escherichia coli* |  |  |
| DH5α | F^-^ 80dlacZ ΔM15 Δ(lacZYA-argF)U169 endA1 recA1 hsdR17(r_K_^-^m_K_^+^) deoR thi-1 supE44 gyrA96 relA1 λ- | Invitrogen, Carlsbad, CA, U.S.A |
| *Erwinia amylovora* |  |  |
| Ea1189 | Wild type | [62] |
| Ea1189∆*hfq* | *hfq* deletion mutant, Cm^r^ | [39] |
| Ea1189∆*ams* | deletion of 12-gene *ams* operon, Cm^r^ | [62] |
| Ea1189∆T3SS | deletion of 24-gene T3SS pathogenicity island, Km^r^ | [62] |
| Ea1189∆*arcZ* (∆*ryhA*) | *arcZ* sRNA deletion mutant, Cm^r^ | [39] |
| Ea1189∆*rprA* | *rprA* sRNA deletion mutant, Cm^r^ | [39] |
| Ea1189∆*spf* | *spf* sRNA deletion mutant, Cm^r^ | [39] |
| Ea1189∆*micA* | *micA* sRNA deletion mutant, Cm^r^ | [39] |
| Ea1189∆*omrAB* | *omrAB* sRNA deletion mutant, Cm^r^ | [39] |
| Ea1189∆*ryhB* | *ryhB* sRNA deletion mutant, Cm^r^ | [39] |
| Ea1189∆*sroB* | *sroB* sRNA deletion mutant, Cm^r^ | [39] |
| Ea1189∆*hrs5* | Hrs5 sRNA deletion mutant, Cm^r^ | This work |
| Ea1189∆*hrs6* | Hrs6 sRNA deletion mutant, Cm^r^ | This work |
| Ea1189∆*hrs8* | Hrs8 sRNA deletion mutant, Cm^r^ | This work |
| Ea1189∆*hrs10* | Hrs10 sRNA deletion mutant, Cm^r^ | This work |
| Ea1189∆*hrs11* | Hrs11 sRNA deletion mutant, Cm^r^ | This work |
| Ea1189∆*hrs12* | Hrs12 sRNA deletion mutant, Cm^r^ | This work |
| Ea1189∆*hrs13* | Hrs13 sRNA deletion mutant, Cm^r^ | This work |
| Ea1189∆*hrs15* | Hrs15 sRNA deletion mutant, Cm^r^ | This work |
| Ea1189∆*hrs20* | Hrs20 sRNA deletion mutant, Cm^r^ | This work |
| Ea1189∆*hrs21* | Hrs21 sRNA deletion mutant, Cm^r^ | This work |
| Ea1189∆*hrs27* | Hrs27 sRNA deletion mutant, Cm^r^ | This work |
| Ea1189∆*hrs29* | Hrs29 sRNA deletion mutant, Cm^r^ | This work |
| Ea1189∆*hrs31* | Hrs31 sRNA deletion mutant, Cm^r^ | This work |
| Ea1189∆*hrs34* | Hrs34 sRNA deletion mutant, Cm^r^ | This work |
| Plasmids |  |  |
| pKD4 | mutagenesis cassette template, Ap^r^, Km^r^ | [49] |
| pKD46 | expresses λ red recombinase, Ap^r^ | [49] |
| pML123 | RSF1010-derived expression and lac-fusion broad-host-range vector, Gm^r^ | [63] |
| pMLhfq | *hfq* with its native promoter cloned at *XbaI/SacI* in pML123, Gm^r^ | [39] |
| pMLarcZ | *arcZ* with its native promoter cloned at *XbaI/SacI* in pML123, Gm^r^ | [39] |
| pMLhrs6 | *rmaA* (*hrs6*) with its native promoter cloned at *XbaI/SacI* in pML123, Gm^r^ | This work |
| pMLomrAB | *omrAB* with its native promoter cloned at *XbaI/SacI* in pML123, Gm^r^ | This work |
|  |  |  |
|  |  |  |

^a^Cm^r^, Gm^r^, and Ap^r^ indicate chloramphenicol, gentamicin, and ampicillin resistance, respectively.

Table S2. Summary of the Rho-independent terminators identified in the genome of *E. amylovora*

| **Genome Location** | **Secondary structure** | **Free energy of secondary structure ∆G (kcal mol^-1^)** | **sRNA upstream?** | **ORF upstream?** |
| --- | --- | --- | --- | --- |
| 52818 | Stem-loop | <-5.0 | Yes (*spf*) |  |
| 99357 | Stem-loop | <-5.0 |  |  |
| 104086 | Stem-loop | <-5.0 |  |  |
| 130143 | Stem-loop | <-5.0 | Yes (*hrs1*) |  |
| 171274 | Stem-loop | <-5.0 |  |  |
| 183883 | Stem-loop | <-5.0 | tRNA |  |
| 187292 | Stem-loop | <-5.0 |  |  |
| 217661 | Stem-loop | <-5.0 | tRNA |  |
| 219038 | Stem-loop | <-5.0 | Yes (*glmZ*) | |
| 236596 | Stem-loop | <-5.0 |  | Yes |
| 245077 | Stem-loop | <-5.0 | Yes (*hrs2*) |  |
| 289286 | Stem-loop | <-5.0 |  | Yes |
| 411632 | Stem-loop | <-5.0 |  | Yes |
| 429227 | Stem-loop | <-5.0 |  | Yes |
| 436613 | Stem-loop | <-5.0 |  |  |
| 528696 | Stem-loop | <-5.0 |  | Yes |
| 554771 | Stem-loop | <-5.0 |  |  |
| 577059 | Stem-loop | <-5.0 |  |  |
| 718384 | Stem-loop | <-5.0 |  |  |
| 834619 | Stem-loop | <-5.0 | Yes (*hrs3*) |  |
| 913263 | Stem-loop | <-5.0 |  | Yes |
| 917829 | Stem-loop | <-5.0 |  |  |
| 922172 | Stem-loop | <-5.0 |  | Yes |
| 964885 | Stem-loop | <-5.0 |  | Yes |
| 1063481 | Stem-loop | <-5.0 |  | Yes |
| 1074149 | Stem-loop | <-5.0 |  | Yes |
| 1116192 | Stem-loop | <-5.0 |  |  |
| 1149296 | Stem-loop | <-5.0 | Yes (*sroB*) |  |
| 1167912 | Stem-loop | <-5.0 |  | Yes |
| 1190675 | Stem-loop | <-5.0 |  |  |
| 1212421 | Stem-loop | <-5.0 |  |  |
| 1213962 | Stem-loop | <-5.0 |  | Yes |
| 1248617 | Stem-loop | <-5.0 |  |  |
| 1250631 | Stem-loop | <-5.0 |  |  |
| 1252249 | Stem-loop | <-5.0 | Yes (*hrs4*) |  |
| 1277604 | Stem-loop | <-5.0 |  |  |
| 1289307 | Stem-loop | <-5.0 |  | Yes |
| 1503414 | Stem-loop | <-5.0 |  |  |
| 1571973 | Stem-loop | <-5.0 |  | Yes |
| 1584270 | Stem-loop | <-5.0 |  |  |
| 1590443 | Stem-loop | <-5.0 |  |  |
| 1598072 | Stem-loop | <-5.0 |  |  |
| 1651318 | Stem-loop | <-5.0 |  |  |
| 1783564 | Stem-loop | <-5.0 | Yes (*hrs6*) |  |
| 1807995 | Stem-loop | <-5.0 |  | Yes |
| 1861357 | Stem-loop | <-5.0 |  | Yes |
| 1964063 | Stem-loop | <-5.0 | Yes (*hrs7*) |  |
| 2019161 | Stem-loop | <-5.0 |  | Yes |
| 2026223 | Stem-loop | <-5.0 |  |  |
| 2059501 | Stem-loop | <-5.0 |  |  |
| 2132831 | Stem-loop | <-5.0 | Yes (*hrs8*) |  |
| 2146157 | Stem-loop | <-5.0 |  |  |
| 2161454 | Stem-loop | <-5.0 |  |  |
| 2201529 | Stem-loop | <-5.0 |  |  |
| 2201714 | Stem-loop | <-5.0 |  |  |
| 2203516 | Stem-loop | <-5.0 |  |  |
| 2203700 | Stem-loop | <-5.0 |  |  |
| 2220881 | Stem-loop | <-5.0 |  |  |
| 2294412 | Stem-loop | <-5.0 |  |  |
| 2302438 | Stem-loop | <-5.0 |  |  |
| 2315491 | Stem-loop | <-5.0 | Yes (*hrs9*) |  |
| 2356768 | Stem-loop | <-5.0 | Yes (*hrs10*) |  |
| 2438169 | Stem-loop | <-5.0 | Yes (*hrs12*) |  |
| 2498520 | Stem-loop | <-5.0 |  |  |
| 2791681 | Stem-loop | <-5.0 |  |  |
| 2822178 | Stem-loop | <-5.0 |  | Yes |
| 2858046 | Stem-loop | <-5.0 |  | Yes |
| 2962947 | Stem-loop | <-5.0 | Yes (*gcvB*) |  |
| 3092922 | Stem-loop | <-5.0 |  | Yes |
| 3344366 | Stem-loop | <-5.0 |  |  |
| 3357244 | Stem-loop | <-5.0 |  |  |
| 3382917 | Stem-loop | <-5.0 |  |  |
| 3399550 | Stem-loop | <-5.0 | Yes (*arcZ*) |  |
| 3573476 | Stem-loop | <-5.0 | Yes (*hrs15*) |  |
| 3672950 | Stem-loop | <-5.0 |  |  |
| 3790675 | Stem-loop | <-5.0 | Yes (*hrs16*) |  |
| 1064009c | Stem-loop | <-5.0 |  | Yes |
| 1120892c | Stem-loop | <-5.0 |  |  |
| 1199184c | Stem-loop | <-5.0 |  | Yes |
| 1213925c | Stem-loop | <-5.0 |  | Yes |
| 1382906c | Stem-loop | <-5.0 |  | Yes |
| 1408436c | Stem-loop | <-5.0 | Yes (*hrs5*) |  |
| 1477636c | Stem-loop | <-5.0 |  | Yes |
| 1479046c | Stem-loop | <-5.0 |  | Yes |
| 156861c | Stem-loop | <-5.0 |  |  |
| 1572126c | Stem-loop | <-5.0 |  | Yes |
| 1705271c | Stem-loop | <-5.0 |  |  |
| 1771835c | Stem-loop | <-5.0 | Yes (*rprA*) |  |
| 1784055c | Stem-loop | <-5.0 |  | Yes |
| 1871174c | Stem-loop | <-5.0 |  | Yes |
| 196242c | Stem-loop | <-5.0 |  | Yes |
| 1981655c | Stem-loop | <-5.0 | Yes (*ryhB*) |  |
| 2008144c | Stem-loop | <-5.0 |  | Yes |
| 2040627c | Stem-loop | <-5.0 |  | Yes |
| 2059465c | Stem-loop | <-5.0 |  | Yes |
| 2117464c | Stem-loop | <-5.0 |  | Yes |
| 2152930c | Stem-loop | <-5.0 |  | Yes |
| 2177336c | Stem-loop | <-5.0 |  | Yes |
| 2181901c | Stem-loop | <-5.0 |  | Yes |
| 2399091c | Stem-loop | <-5.0 | Yes (*hrs11*) |  |
| 2445993c | Stem-loop | <-5.0 |  |  |
| 2580017c | Stem-loop | <-5.0 |  |  |
| 2610754c | Stem-loop | <-5.0 | Yes (*hrs13*) |  |
| 2728844c | Stem-loop | <-5.0 |  |  |
| 2917993c | Stem-loop | <-5.0 |  |  |
| 2942062c | Stem-loop | <-5.0 |  |  |
| 3009347c | Stem-loop | <-5.0 | Yes (*omrAB*) |  |
| 3037320c | Stem-loop | <-5.0 |  | Yes |
| 3075650c | Stem-loop | <-5.0 |  | Yes |
| 3357214c | Stem-loop | <-5.0 |  |  |
| 3369436c | Stem-loop | <-5.0 |  |  |
| 340730c | Stem-loop | <-5.0 |  | Yes |
| 3473001c | Stem-loop | <-5.0 |  |  |
| 3572614c | Stem-loop | <-5.0 |  | Yes |
| 3794902c | Stem-loop | <-5.0 |  | Yes |
| 745091c | Stem-loop | <-5.0 |  | Yes |
| 799893c | Stem-loop | <-5.0 |  |  |

Table S3. The percentage of sRNAs in the total sRNA pool in the wild type Ea1189 at 6 and 12 hrs post induction in Hrp-inducing minimal medium

| **sRNA ID** | **6hr** | **12hr** |
| --- | --- | --- |
| Hrs12 | 29.5 | 35.7 |
| ArcZ (RyhA) | 7.5 | 10.5 |
| Spf (Spot42) | 6.0 | 8.0 |
| Hrs1 | 5.8 | 1.1 |
| RprA | 4.2 | 4.6 |
| Hrs4 | 3.8 | 0.8 |
| Hrs13 | 3.8 | 3.7 |
| Hrs31 | 3.7 | 2.8 |
| Hrs21 | 3.0 | 4.4 |
| Hrs9 | 2.1 | 1.5 |
| Hrs5 | 1.5 | 1.2 |
| Hrs19 | 1.5 | 0.4 |
| Hrs20 | 1.4 | 1.4 |
| Hrs11 | 1.1 | 1.2 |
| Hrs2 | 1.0 | 0.5 |
| Hrs6 | 1.0 | 0.7 |
| Hrs17 | 0.8 | 0.6 |
| Hrs8 | 0.7 | 1.7 |
| GcvB | 0.7 | 4.5 |
| Hrs10 | 0.5 | 0.7 |
| Hrs27 | 0.5 | 0.4 |
| Hrs23 | 0.4 | 1.8 |
| RyhB | 0.4 | 0.7 |
| MicA | 0.3 | 0.6 |
| Hrs18 | 0.3 | 0.2 |
| OmrAB | 0.3 | 0.2 |
| Hrs24 | 0.3 | 0.1 |
| Hrs29 | 0.2 | 0.1 |
| Hrs34 | 0.2 | 0.1 |
| Hrs15 | 0.2 | 0.1 |
| Hrs28 | 0.2 | 0.3 |
| Hrs33 | 0.2 | 0.3 |
| Hrs30 | 0.1 | 0.3 |
| Hrs7 | 0.1 | 0.1 |
| Hrs32 | 0.1 | 0.3 |
| Hrs26 | 0.1 | 0.0 |
| MicM (SroB) | 0.1 | 0.0 |
| Hrs25 | 0.1 | 0.0 |
| Hrs16 | 0.1 | 0.0 |
| Hrs3 | 0.0 | 0.0 |
|  |  |  |
